# Heart Under Pressure: Divergent Cardiac Molecules Responses to Azathioprine and Anti-TNF Therapy in Ulcerative Colitis

**DOI:** 10.3390/ijms262010160

**Published:** 2025-10-19

**Authors:** Mirjana Cvetković, Stefan Simović, Dušan Radojević, Mladen Maksić, Nataša Zdravković, Bojan Milošević, Bojan Stojanović, Radojica Stolić, Željko Todorović, Anđela Gogić, Nebojša Zdravković, Mateja Zdravković

**Affiliations:** 1 Department of Gastroenterology and Hepatology, University Hospital Medical Center Bežanijska kosa, 11080 Belgrade, Serbia; mirjana.cvetkovic71@gmail.com; 2 Department of Internal Medicine, Faculty of Medical Sciences, University of Kragujevac, 34000 Kragujevac, Serbiaasussonicmaster95@gmail.com (M.M.); natasasilvester@gmail.com (N.Z.); radojicastolic61@gmail.com (R.S.); todorovic_zeljko@hotmail.com (Ž.T.); 3 Clinic for Cardiology, University Clinical Center Kragujevac, 34000 Kragujevac, Serbia; 4 Clinic for Gastroenterology and Hepatology, University Clinical Center Kragujevac, 34000 Kragujevac, Serbia; 5 Department of Surgery, Faculty of Medical Sciences, University of Kragujevac, 34000 Kragujevac, Serbia; drbojanzm@gmail.com (B.M.); bojan.stojanovic01@gmail.com (B.S.); 6 Clinic for General Surgery, University Clinical Center Kragujevac, 34000 Kragujevac, Serbia; 7 Clinic for Nephrology and Dialysis, University Clinical Center Kragujevac, 34000 Kragujevac, Serbia; 8 Clinic for Hematology, University Clinical Center Kragujevac, 34000 Kragujevac, Serbia; 9 Department of Medical Statistics and Informatics, Faculty of Medical Sciences, University of Kragujevac, 34000 Kragujevac, Serbia; andjelica97@hotmail.com (A.G.); nzdravkovic@gmail.com (N.Z.); mzdravkovic39@gmail.com (M.Z.)

**Keywords:** ulcerative colitis, adalimumab, infliximab, azathioprine, proBNP, NT-proBNP, long-term therapy

## Abstract

The treatment of moderate-to-severe ulcerative colitis often requires escalation from 5-ASA therapy to immunosuppressants or biologic agents. However, the effects of azathioprine and anti-TNF therapies on cardiac status and associated biomolecules remain insufficiently studied. In this prospective observational study, we included 84 patients with moderate-to-severe ulcerative colitis, divided into three treatment groups based on the therapy received (azathioprine, infliximab, or adalimumab), along with 25 healthy controls. Levels of proBNP, NT-proBNP, creatine kinase (CK), CK-MB, and high-sensitivity troponin I (hsTnI) were measured at baseline, 6 h after treatment initiation, and after 3 months of therapy. Troponin levels did not significantly change across the three time points in any treatment group. In contrast, levels of proBNP, NT-proBNP, CK, and CK-MB significantly decreased after 3 months in patients treated with biologics, whereas a significant increase was observed in the azathioprine group. Further prospective trials are needed to adequately assess the cardiac safety of these therapies, particularly in patients with preexisting cardiac impairment.

## 1. Introduction

Ulcerative colitis (UC) is an autoimmune disease of the colon characterized by inflammation that begins in the rectum and extends proximally in a continuous pattern. This subtype of inflammatory bowel disease (IBD) is confined to the mucosa of the affected colon, presenting with visible macroscopic features such as erythema, erosions, and ulcerations, often accompanied by spontaneous bleeding. Diagnosis is based on clinical symptoms, radiological and laboratory findings, and is confirmed by colonoscopy with biopsy. Patients with UC commonly experience rectal bleeding, loose or watery stools, bowel urgency, nocturnal bowel movements, and abdominal pain [1]. Typical endoscopic findings include edema, loss of vascularity, mucosal granularity, erythema, erosions, ulcerations, and pseudopolyps [2]. A definitive diagnosis is made through histopathological examination, with current guidelines recommending biopsies from each segment of the colon [3]. Because there is no single pathognomonic sign, diagnosis requires the exclusion of infectious causes along with the presence of typical histological features. Histopathology is consistent with chronic active colitis, assessed by the presence of neutrophils in the surface epithelium, cryptitis (neutrophils within crypt epithelium), or crypt abscesses (collections of neutrophils within crypt lumens) [4]. Eosinophil infiltration and basal plasmacytosis further support a diagnosis of UC [5]. Long-standing disease is marked by crypt distortion and Paneth cell metaplasia in the left colon and rectum [6]. Extraintestinal manifestations of UC are common, with arthritis being the most prevalent [1]. Cardiovascular disease (CVD), although less frequent, represents a clinically significant extraintestinal manifestation of IBD. The association between IBD and CVD is well documented and attributed to chronic inflammation and elevated levels of the proinflammatory cytokine tumor necrosis factor alpha (TNF-α), indicating that IBD is an independent risk factor for CVD [7,8]. UC patients are at increased risk of ischemic heart disease, with the highest CVD-related mortality observed in the first year following diagnosis [9,10]. Compared to the general population, UC patients are also more likely to develop heart failure, particularly younger patients and females [11,12]. Chronic systemic inflammation is believed to drive atherogenesis and thrombogenesis in various inflammatory diseases [13]. An echocardiographic study in patients with Crohn’s disease reported frequent morphological abnormalities, especially mitral valve prolapse and pericardial effusion [14]. Since TNF-α plays a role in atherosclerosis, controlling inflammation in IBD remains a critical strategy to reduce cardiovascular risk [15,16]. In mild-to-moderate UC, remission is achieved using topical and systemic 5-aminosalicylates, while moderate to severe cases often require escalation to immunosuppressants or biologic therapies. Azathioprine (AZA) is the most commonly used immunosuppressant, while infliximab (IFX, a chimeric monoclonal antibody) and adalimumab (ADA, a fully human monoclonal anti-TNF-α antibody) are the most frequently prescribed biologics [1]. N-terminal pro–B–type natriuretic peptide (NT-proBNP) is a well-established biomarker of myocardial wall stress and neurohormonal activation, widely used in the diagnosis, risk stratification, and monitoring of heart failure [17]. It also serves as a sensitive marker of subclinical ventricular dysfunction and cardiovascular risk in various clinical settings [18]. In patients with coronary artery disease, an increase in NT-proBNP levels over a 6-month period was associated with an elevated risk of cardiovascular death or hospitalization for heart failure [19].

Although limited, existing studies suggest that elevated NT-proBNP levels in IBD patients correlate with endoscopic, histologic, and clinical disease activity, indicating potential utility as a biomarker for UC activity [20].

## 2. Results

**Figure 1:**
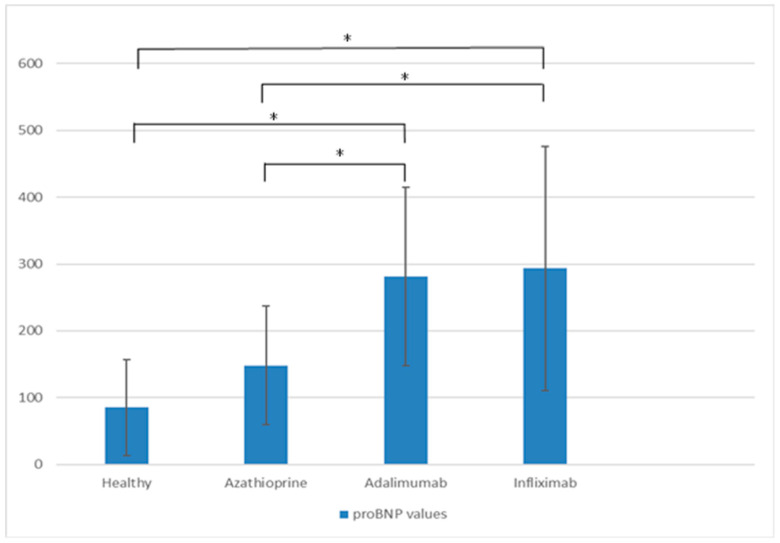
proBNP values at baseline across the four groups. Asterisks denote statistically significant differences. After correction, the significance level was set at *p* < 0.0083.

All three patient groups had higher baseline proBNP levels compared to healthy controls, although statistical significance was reached only in the biologic treatment groups (Figure 1). On initial evaluation, the AZA group had fewer patients with a severe Mayo endoscopic score (*n* = 3) than the IFX (*n* = 13) or ADA (*n* = 11) groups. Compared to IFX and ADA, the AZA group also showed lower histologic scores, fecal calprotectin, troponin, NT-proBNP, and proBNP values, as presented in Table 1.

### 2.1. Short-Term Effects

Six hours after administration of AZA, IFX, and ADA, mean NT-proBNP values increased by 102, 72, and 23, respectively (Table 2). All differences reached statistical significance, and similar results were observed for proBNP. Additionally, troponin levels increased significantly six hours after administration only in the AZA group (mean value: 0.0039, *p* = 0.000). The highest absolute values of proBNP and NT-proBNP at 6 h were observed in the IFX group, while the AZA group demonstrated the greatest relative increase from baseline.

### 2.2. Long-Term Effects

**Figure 2:**
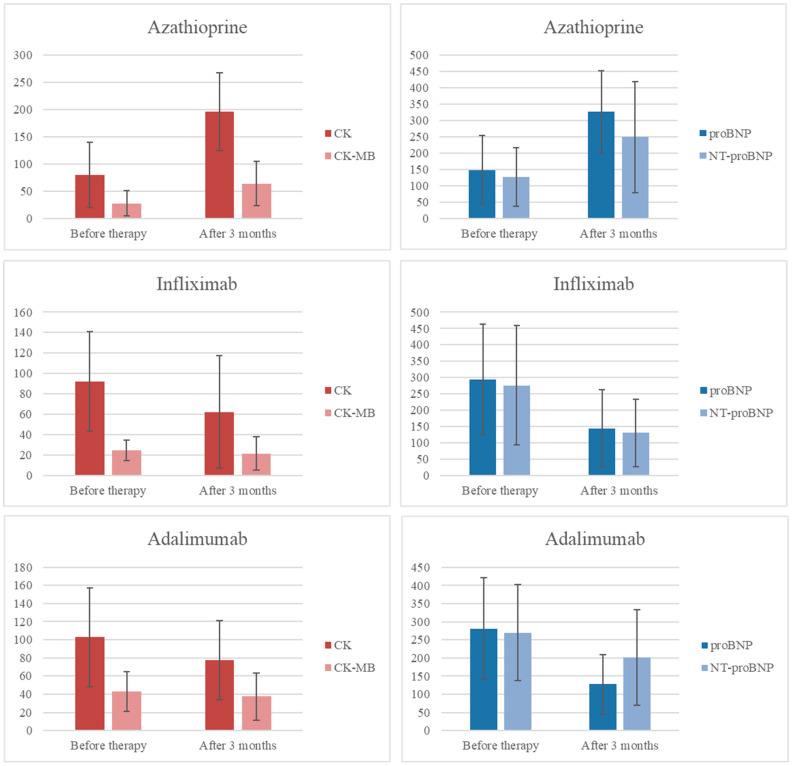
Comparison of proBNP, NT-proBNP, CK and CK-MB across the three treatment groups (AZA, IFX, ADA) was performed. Because proBNP and NT-proBNP were measured at three time points, a Bonferroni correction for multiple time point comparisons was applied (α = 0.05/3 = 0.0167); for CK and CK-MB, which were assessed at two time points, the conventional significance threshold of *p* < 0.05 was used. ProBNP, NT-proBNP, CK and CK-MB decreased in both biologic groups and increased in the azathioprine group. All changes reached statistical significance under the specified thresholds except CK-MB in the IFX group (*p* = 0.069), and NT-proBNP in the ADA group (*p* = 0.021), which did not meet the Bonferroni-adjusted cutoff.

After three months of therapy, levels of proBNP, NT-proBNP, CK, and CK-MB were significantly lower in both biologic therapy groups compared to the AZA group, while the troponin levels remained unchanged, as shown in Table 3. A comparison of cardiac biomarkers at baseline and after three months of therapy (Figure 2) revealed that both anti-TNF agents significantly reduced proBNP and NT-proBNP levels (*p* = 0.001 for both markers in the infliximab group; *p* = 0.000 for proBNP in the adalimumab group). Although NT-proBNP levels also decreased in the adalimumab group, the change was not statistically significant. Troponin levels did not change in any treatment group. In contrast, patients receiving azathioprine showed significant increases in proBNP, NT-proBNP, CK, and CK-MB levels after three months of therapy (*p* = 0.000 for all markers). The largest reduction in cardiac biomarkers was observed in the infliximab group.

## 3. Discussion

The significance of natriuretic peptides in cardiovascular disease is unparalleled. NT-proBNP is used in diagnosis, treatment selection, and prognosis of heart failure patients [21]. As previously mentioned, its dynamics are a supreme marker of cardiovascular disease progression [19]. Our study, together with previous research, underscores the need for further investigation into the role of natriuretic peptides in IBD, particularly to determine whether timely serial measurements of NT-proBNP or proBNP could provide a clinical benefit in guiding therapy.

### 3.1. Short-Term Effects on Cardiac Biomarkers

Baseline proBNP values in our study are consistent with previous findings indicating mildly elevated proBNP levels in patients with severe UC compared to healthy controls. Differences observed in baseline cardiac biomarker levels between the AZA and anti-TNF groups likely reflect differences in disease severity. Patients treated with anti-TNF agents had higher levels of laboratory markers of intestinal inflammation; more severe histologic findings; and overall, more advanced disease. Our findings replicate and reinforce previously documented acute effects of infliximab administration. In a study by Tomáš et al. [22], plasma proBNP levels were measured at 6 and 12 months after IFX therapy, showing a decrease similar to what we observed, although their results did not reach statistical significance—likely due to the small sample size. They also assessed echocardiographic features in all time points but found no statistical differences [22].

To our knowledge, this is the first study to directly compare the acute effects of in-fliximab, adalimumab, and azathioprine on cardiac biomarkers such as proBNP, creatine kinase, and troponin. Although both anti-TNF agents caused a statistically significant increase in natriuretic peptide levels shortly after administration, the response was less pronounced with adalimumab. This may be explained by the greater cytotoxic potential of infliximab, its intravenous route of administration (versus subcutaneous for adalimumab), or by the fluid load from concurrent saline infusion during IFX therapy [22]. While previous reports indicate that 250 mL of saline has only a modest effect on natriuretic peptides [23], standard protocol at our center involves the administration of infliximab in 500 mL of saline, which may cause a higher increase in circulating natriuretic peptide levels.

### 3.2. Long-Term Use of Immunosuppressants and Anti-TNF Agents: Future Implications

#### 3.2.1. Azathioprine

Azathioprine has been used for more than five decades as maintenance therapy in IBD, with well-established steroid-sparing efficacy [24,25]. Adverse effects occur in approximately 15–30% of patients and frequently lead to discontinuation. While dose-dependent myelosuppression and hepatotoxicity can often be predicted through *TPMT* and *NUDT15* pharmacogenetic testing, gastrointestinal intolerance and acute pancreatitis remain common. Long-term use of azathioprine is associated with a slightly increased risk of opportunistic infections and malignancies; patients on AZA therapy have a two- to four-fold increased risk of non-melanoma skin cancer and non-Hodgkin’s lymphoma compared to the general population [26,27]. Cardiotoxicity associated with azathioprine is exceedingly rare and poorly understood, with current evidence limited to case reports and small case series. Our study demonstrated a statistically significant increase in cardiac biomarkers after three months of AZA therapy, in contrast to the decrease seen with anti-TNF treatment. Hypersensitivity myocarditis and pericarditis have been described in the context of systemic hypersensitivity reactions to azathioprine, typically occurring within days to weeks of therapy initiation and resolving upon drug withdrawal. For example, a case of acute pericarditis presenting with chest pain, fever, and ECG changes in a young man with UC resolved completely after stopping azathioprine, while continuing mesalamine [28]. Similarly, cases of myocarditis with chest pain, dyspnea, and elevated cardiac enzymes have been reported, likely due to immune-mediated mechanisms rather than direct cardiotoxicity [29]. A 2012 case report described cardiogenic shock following its administration [30]. Another case linked azathioprine to ST-segment elevation myocardial infarction and distributive shock one week after therapy initiation, as part of the so-called azathioprine hypersensitivity syndrome [31,32]. Rare cases of arrhythmia have also been documented, including one instance of atrial fibrillation in a middle-aged man with UC, which resolved upon discontinuation of azathioprine and did not recur with alternative treatment [33]. However, most of these cardiac side effects are acute, idiosyncratic, and based on isolated reports. They do not account for the consistent elevation of proBNP observed after three months of AZA therapy in our study population. Our interpretation of the available evidence suggests two potential mechanisms: the formation of reactive oxygen species (ROS) in cardiac tissue and inadequate disease control. Both, however, seem unlikely. The former is speculative and has not been demonstrated in cardiac tissue; the latter would imply that patients treated with AZA had worsening disease after three months, which contradicts current clinical guidelines and long-standing experience with azathioprine as an effective maintenance agent [24,25]. Experimental cell culture studies have shown that azathioprine-induced hepatotoxicity and cytotoxicity are mediated primarily through the generation of ROS and mitochondrial dysfunction. While these mechanisms have not been confirmed in cardiac tissue, theoretical cardiomyocyte susceptibility cannot be entirely ruled out without further investigation [34,35]. Our study demonstrates a correlation and suggests a potential causal relationship between azathioprine use and elevated cardiac biomarker levels. However, we did not measure azathioprine levels or its metabolites and further research—including prospective clinical trials and mechanistic studies—is needed to better understand this association. To the best of our knowledge, these findings are novel in the context of azathioprine therapy and warrant further scientific exploration.

#### 3.2.2. Infliximab and Adalimumab

Recognition of TNF’s role in the pathogenesis of heart failure led to early experimental trials in the 2000s that tested anti-TNF agents as a therapeutic option. However, these studies were halted after showing increased mortality [36]. To this day, infliximab remains contraindicated in patients with NYHA class III and IV heart failure [37]. It is important to emphasize that the recommendation is based on this pivotal study which involved patients with NYHA class III and IV heart failure who did not have concomitant inflammatory diseases such as ulcerative colitis or rheumatoid arthritis, diseases known for their elevated TNF-α levels. Moreover, the harmful dosage in that study was 10 mg/kg, while the standard dose used in ulcerative colitis is typically 5 mg/kg—an amount shown to have effects comparable to placebo. The safety and effects of infliximab in patients with NYHA class I and II heart failure remain poorly studied [37]. Despite isolated case reports of cardiac adverse events, long-term anti-TNF therapy has not been shown to impair myocardial function. Patients without preexisting cardiac disease receiving anti-TNF agents exhibit a very low risk of developing heart failure [38,39,40,41,42,43]. In contrast, it is well-established that effective disease control reduces cardiovascular risk in patients with ulcerative colitis. Studies have reported a significant reduction in the risk of ischemic heart disease (IHD) in patients treated with 5-aminosalicylates, and a modest risk reduction in those receiving azathioprine or anti-TNF therapy [44]. Furthermore, a randomized controlled trial published two years ago demonstrated that anti-TNF therapy may offer superior cardiovascular protection in patients with rheumatologic inflammatory diseases at high cardiovascular risk compared to other treatments [45]. In our study, levels of cardiac biomarkers—including CK-MB, proBNP, and NT-proBNP—decreased following three months of anti-TNF therapy, likely reflecting improved disease control. In addition to being considered safe from a cardiovascular standpoint, recent studies have also suggested that anti-TNF therapy may have cardioprotective effects [46,47]. Presently, anti-TNF agents are increasingly being explored as potential treatments for various cardiac conditions. Notably, a recent study published in JAHA identified TNF-α as a key mediator of myocardial infarction; infliximab infusion in an experimental porcine model led to reduced myocardial injury and improved cardiac recovery [48]. These findings raise the possibility that infliximab and adalimumab may offer protective effects on the myocardium, challenging the presumption that they are harmful in patients with early-stage heart failure. However, a limitation of our study is the lack of echocardiographic and clinical cardiac assessments, indicating that our findings capture biochemical rather than structural or functional aspects of cardiac involvement. This underscores the importance of further research into the management of patients with both inflammatory bowel disease and NYHA class I or II heart failure and potentially even select patients with more advanced disease (NYHA class III or IV).

## 4. Materials and Methods

This study was designed as a clinical, observational, study conducted at the University Clinical Center Kragujevac, Clinic for Gastroenterology and Hepatology, in collaboration with the Faculty of Medical Sciences, University of Kragujevac. The study was approved by the institutional ethics committees, and all participants provided written informed consent prior to enrolment. Researchers adhered to the principles of Good Clinical Practice throughout the study. Study power and sample size were calculated using G*Power version 3.1. The sample size estimation was based on a previously published and similarly designed study reporting carotid–radial pulse wave velocity values that compared patients with ulcerative colitis and healthy controls [38]. An independent two-sample Student’s t-test (two-tailed) was used with α = 0.05 and power (1 − β) = 0.8. The minimum required sample size was 10 subjects per group (30 subjects in total).

A total of 109 participants were enrolled, including 84 patients with moderate to-severe ulcerative colitis and 25 healthy controls (14 male). UC patients were assigned to one of three treatment groups: infliximab (*n* = 28, 20 male), adalimumab (*n* = 28, 14 male), or azathioprine (*n* = 28, 8 male). Prior to the study, azathioprine patients were screened for the *TPMT* mutation, and only patients with full TPMT activity have been enrolled. Patients in the infliximab group received IFX as monotherapy. All patients were anti-TNF naïve at baseline and were treated with standard doses of mesalamine prior to initiation of biologic therapy.

Inclusion criteria for the experimental group required a confirmed diagnosis of ulcerative colitis, established through endoscopic examination and histopathological analysis of colonic mucosal biopsies, in accordance with the criteria outlined in the 2017 Third European Evidence-Based Consensus on the Diagnosis and Management of Ulcerative Colitis [3]. Control group participants were required to be healthy individuals with no known inflammatory, cardiovascular, or malignant conditions. All participants were required to voluntarily provide written informed consent prior to enrolment. Additionally, UC patients had to meet all clinical criteria for initiating biologic or immunosuppressive therapy, according to standard treatment guidelines. Exclusion criteria included any prior diagnosis of cardiovascular disease (e.g., myocardial infarction, unstable angina pectoris, any history of heart failure, hypertensive crisis within the past month, or previous stroke or transient ischemic attack). Patients with chronic renal failure requiring dialysis at any point were also excluded. Additional exclusion criteria included age under 18 years, pregnancy or breastfeeding, cognitive impairment, or legally limited decision-making capacity. Participants were also excluded if they had an active infectious syndrome or any infection within two months prior to enrolment or during the study period. Chronic or malignant diseases, as well as the use of medications that could directly or indirectly affect study parameters, were further grounds for exclusion.

All UC patients underwent lower endoscopic examination before initiating therapy. Disease activity was assessed using the Montreal classification, the Truelove and Witts severity index, the Mayo endoscopic subscore, and the Geboes histological score. After baseline laboratory evaluation, patients began treatment with their assigned therapeutic regimen—IFX, ADA, or AZA—administered in accordance with standard induction protocols. Blood samples were collected at three defined time points: (1) at baseline before treatment initiation, (2) six hours after the first drug administration, and (3) three months after therapy initiation. Samples were collected into EDTA vacutainer tubes and serum tubes and centrifuged accordingly. Baseline laboratory analyses included CBC, ESR, CRP, proBNP, NT-proBNP, high-sensitivity troponin I, CK, CK-MB, serum iron, and ferritin. Six hours after the first drug dose, additional blood samples were collected for proBNP, NT-proBNP, and high-sensitivity troponin I, which were reassessed again three months later, along with CK and CK-MB. All laboratory tests were performed in the Biochemical Laboratory of the University Clinical Center Kragujevac. Statistical analysis was conducted using IBM SPSS software, version 20. Normality of data distribution was assessed using the Shapiro–Wilk test. Due to the non-normal distributions, the Kruskal–Wallis test was used to evaluate differences among the three independent groups. Post hoc pairwise comparisons were conducted using the Mann–Whitney U test with appropriate correction for multiple testing. The threshold for statistical significance was set at *p* < 0.0167.

## 5. Conclusions

Anti-TNF agents play a crucial role in the management of severe ulcerative colitis. Our findings point to two clinical considerations: a potential rationale for a more permissive attitude toward biologic therapy (including anti-TNF agents) in patients with heart failure, and the need for particular caution when contemplating azathioprine in the same setting. In this context, molecular biomarkers, such as natriuretic peptides, may play a pivotal role in therapeutic decision-making. These are preliminary, hypothesis-generating observations that call for well-designed, prospective studies with adequate power and systematic cardiovascular assessment to establish safety profiles and inform optimal treatment choices for patients with coexisting inflammatory bowel disease and cardiovascular disease; if corroborated, our results should prompt increased caution in selecting azathioprine for those at elevated cardiovascular risk.

## Figures and Tables

**Table 1:** Baseline values of pertinent biomarkers in the three treatment groups. The *p* value threshold was set at <0.0167 after adequate Bonferroni correction.

	Azathioprine	Adalimumab	Infliximab	*p* Value	
				vs. ADA	vs. IFX
proBNP (pg/mL)	148.29	281.38	293.68	0.000	0.001
NT-proBNP (pg/mL)	126.83	270.13	276.32	0.000	0.000
Troponin (ng/mL)	0.001	0.0045	0.0067	0.000	0.000
CK (U/L)	80.33	102.67	92.04	NS	NS
CK-MB (U/L)	27.96	43.00	24.57	0.004	NS
CRP (mg/L)	101.28	58.92	70.73	NS	NS
FCP (µg/g)	486.65	1000.73	1522	0.001	0.015
SE (mm/h)	18.58	36.58	41.28	0.006	0.005
Histologic score *	5.2	5.3	5.3	0.001	0.000

* Median values shown. NS—not significant.

**Table 2:** Values of natriuretic peptides measured six hours after administration of each drug. Statistically significant increases were observed across all groups compared to baseline levels of proBNP and NT-proBNP.

	Azathioprine	Adalimumab	Infliximab
proBNP 6 h (pg/mL)	244.66	311.45	351.39
NT-proBNP 6 h (pg/mL)	229.04	293.95	348.82

**Table 3:** Values of cardiac markers three months after therapy initiation. *p* < 0.0167 was set as a threshold for statistical significance for proBNP, NT-proBNP and troponin. We have set the standard threshold for statistical significance of *p* < 0.05 for CK and CK-MB. 3 M—after three months of therapy. NS—not significant.

	Azathioprine	Adalimumab	Infliximab	*p* Value	
				vs. ADA	vs. IFX
proBNP 3 M (pg/mL)	326.38	127.86	144.86	0.000	0.000
NT-proBNP 3 M (pg/mL)	248.88	201.5	130.6	NS	0.000
Troponin 3 M (ng/mL)	0.001	0.0073	0.009	0.000	0.000
CK 3 M (U/L)	196.75	77.12	62.18	0.000	0.000
CK-MB 3 M (U/L)	64.67	37.34	21.54	0.012	0.000

## Data Availability

The data presented in this study are available on request from the corresponding author.
